# Supplementary figures and images for: Identification of key miRNAs in the progression of hepatocellular carcinoma using an integrated bioinformatics approach
Source: PeerJ. 2020 May 6;8:e9000. doi: 10.7717/peerj.9000 (PMC7210814; doi:10.7717/peerj.9000)

mRNAs in co-expression module

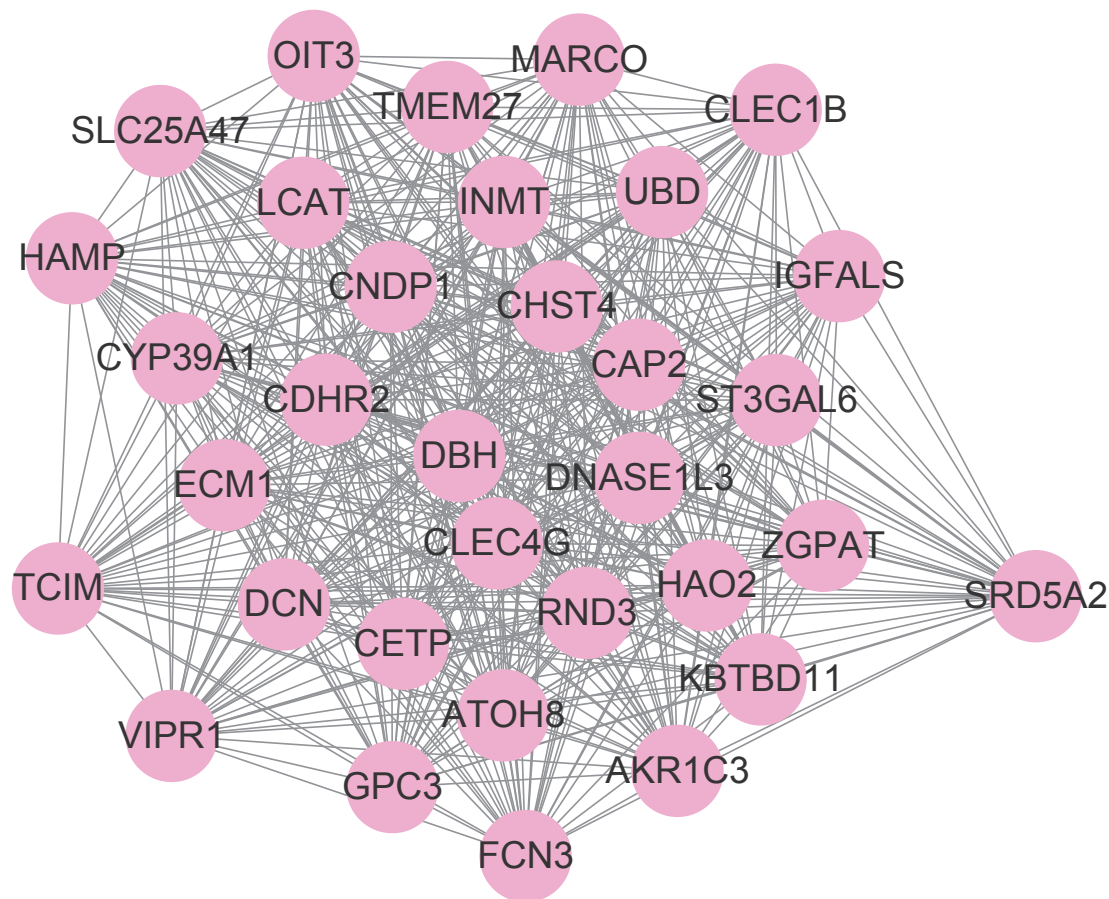

Supplement: Supplemental Information 1 [file peerj-08-9000-s001.pdf]
