# Supplementary material for: Identification of key miRNAs in the progression of hepatocellular carcinoma using an integrated bioinformatics approach
Source: PeerJ. 2020 May 6;8:e9000. doi: 10.7717/peerj.9000 (PMC7210814; doi:10.7717/peerj.9000)
Supplement: Supplemental Information 2 [file peerj-08-9000-s002.pdf]

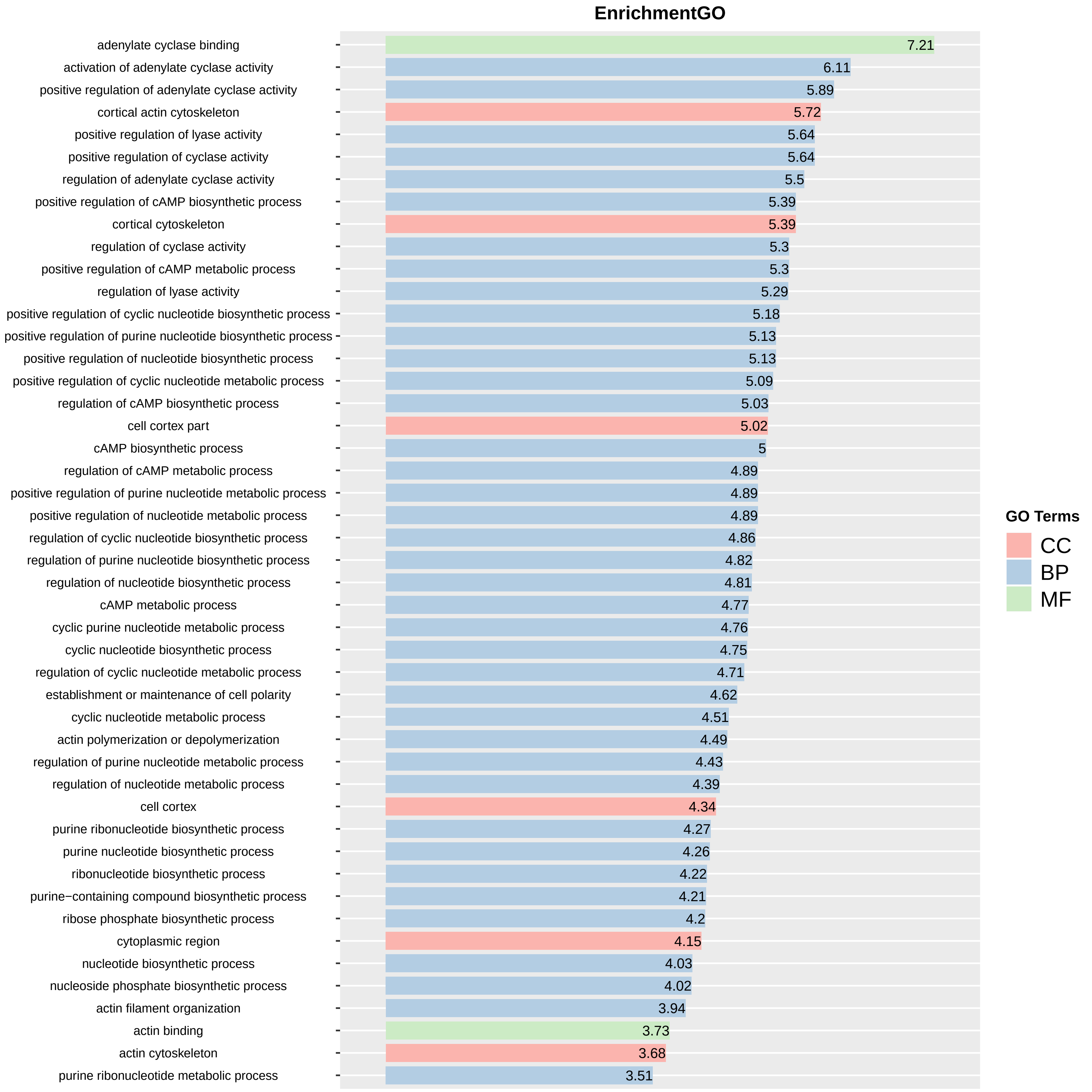
EnrichmentGO
adenylate cyclase binding 7.21
activation of adenylate cyclase activity 6.11
positive regulation of adenylate cyclase activity 5.89
cortical actin cytoskeleton 5.72
positive regulation of lyase activity 5.64
positive regulation of cyclase activity 5.64
regulation of adenylate cyclase activity 5.5
positive regulation of cAMP biosynthetic process 5.39
cortical cytoskeleton 5.39
regulation of cyclase activity 5.3
positive regulation of cAMP metabolic process 5.3
regulation of lyase activity 5.29
positive regulation of cyclic nucleotide biosynthetic process 5.18
positive regulation of purine nucleotide biosynthetic process 5.13
positive regulation of nucleotide biosynthetic process 5.13
positive regulation of cyclic nucleotide metabolic process 5.09
regulation of cAMP biosynthetic process 5.03
cell cortex part 5.02
cAMP biosynthetic process 5
regulation of cAMP metabolic process 4.89
positive regulation of purine nucleotide metabolic process 4.89
positive regulation of nucleotide metabolic process 4.89
regulation of cyclic nucleotide biosynthetic process 4.86
regulation of purine nucleotide biosynthetic process 4.82
regulation of nucleotide biosynthetic process 4.81
cAMP metabolic process 4.77
cyclic purine nucleotide metabolic process 4.76
cyclic nucleotide biosynthetic process 4.75
regulation of cyclic nucleotide metabolic process 4.71
establishment or maintenance of cell polarity 4.62
cyclic nucleotide metabolic process 4.51
actin polymerization or depolymerization 4.49
regulation of purine nucleotide metabolic process 4.43
regulation of nucleotide metabolic process 4.39
cell cortex 4.34
purine ribonucleotide biosynthetic process 4.27
purine nucleotide biosynthetic process 4.26
ribonucleotide biosynthetic process 4.22
purine−containing compound biosynthetic process 4.21
ribose phosphate biosynthetic process 4.2
cytoplasmic region 4.15
nucleotide biosynthetic process 4.03
nucleoside phosphate biosynthetic process 4.02
actin filament organization 3.94
actin binding 3.73
actin cytoskeleton 3.68
purine ribonucleotide metabolic process 3.51
GO Terms
CC
BP
MF

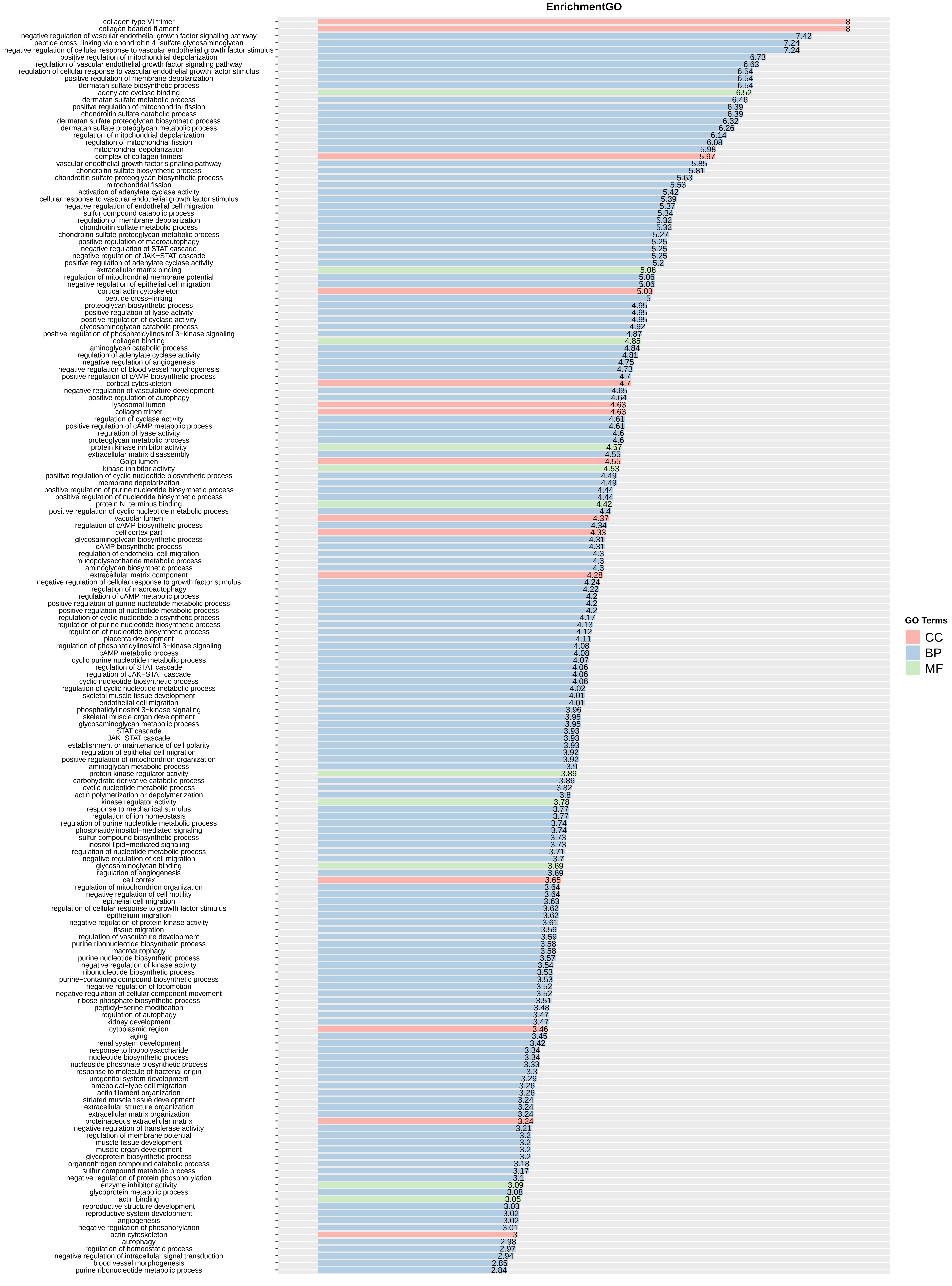
EnrichmentGO
collagen type VI trimer 8
collagen beaded filament 8
negative regulation of vascular endothelial growth factor signaling pathway 7.42
peptide cross−linking via chondroitin 4−sulfate glycosaminoglycan 7.24
negative regulation of cellular response to vascular endothelial growth factor stimulus 7.24
positive regulation of mitochondrial depolarization 6.73
regulation of vascular endothelial growth factor signaling pathway 6.63
regulation of cellular response to vascular endothelial growth factor stimulus 6.54
positive regulation of membrane depolarization 6.54
dermatan sulfate biosynthetic process 6.54
adenylate cyclase binding 6.52
dermatan sulfate metabolic process 6.46
positive regulation of mitochondrial fission 6.39
chondroitin sulfate catabolic process 6.39
dermatan sulfate proteoglycan biosynthetic process 6.32
dermatan sulfate proteoglycan metabolic process 6.26
regulation of mitochondrial depolarization 6.14
regulation of mitochondrial fission 6.08
mitochondrial depolarization 5.98
complex of collagen trimers 5.97
vascular endothelial growth factor signaling pathway 5.85
chondroitin sulfate biosynthetic process 5.81
chondroitin sulfate proteoglycan biosynthetic process 5.63
mitochondrial fission 5.53
activation of adenylate cyclase activity 5.42
cellular response to vascular endothelial growth factor stimulus 5.39
negative regulation of endothelial cell migration 5.37
sulfur compound catabolic process 5.34
regulation of membrane depolarization 5.32
chondroitin sulfate metabolic process 5.32
chondroitin sulfate proteoglycan metabolic process 5.27
positive regulation of macroautophagy 5.25
negative regulation of STAT cascade 5.25
negative regulation of JAK−STAT cascade 5.25
positive regulation of adenylate cyclase activity 5.2
extracellular matrix binding 5.08
regulation of mitochondrial membrane potential 5.06
negative regulation of epithelial cell migration 5.06
cortical actin cytoskeleton 5.03
peptide cross−linking 5
proteoglycan biosynthetic process 4.95
positive regulation of lyase activity 4.95
positive regulation of cyclase activity 4.95
glycosaminoglycan catabolic process 4.92
positive regulation of phosphatidylinositol 3−kinase signaling 4.87
collagen binding 4.85
aminoglycan catabolic process 4.84
regulation of adenylate cyclase activity 4.81
negative regulation of angiogenesis 4.75
negative regulation of blood vessel morphogenesis 4.73
positive regulation of cAMP biosynthetic process 4.7
cortical cytoskeleton 4.7
negative regulation of vasculature development 4.65
positive regulation of autophagy 4.64
lysosomal lumen 4.63
collagen trimer 4.63
regulation of cyclase activity 4.61
positive regulation of cAMP metabolic process 4.61
regulation of lyase activity 4.6
proteoglycan metabolic process 4.6
protein kinase inhibitor activity 4.57
extracellular matrix disassembly 4.55
Golgi lumen 4.55
kinase inhibitor activity 4.53
positive regulation of cyclic nucleotide biosynthetic process 4.49
membrane depolarization 4.49
positive regulation of purine nucleotide biosynthetic process 4.44
positive regulation of nucleotide biosynthetic process 4.44
protein N−terminus binding 4.42
positive regulation of cyclic nucleotide metabolic process 4.4
vacuolar lumen 4.37
regulation of cAMP biosynthetic process 4.34
cell cortex part 4.33
glycosaminoglycan biosynthetic process 4.31
cAMP biosynthetic process 4.31
regulation of endothelial cell migration 4.3
mucopolysaccharide metabolic process 4.3
aminoglycan biosynthetic process 4.3
extracellular matrix component 4.28
negative regulation of cellular response to growth factor stimulus 4.24
regulation of macroautophagy 4.22
regulation of cAMP metabolic process 4.2
positive regulation of purine nucleotide metabolic process 4.2
positive regulation of nucleotide metabolic process 4.2
regulation of cyclic nucleotide biosynthetic process 4.17
regulation of purine nucleotide biosynthetic process 4.13
regulation of nucleotide biosynthetic process 4.12
placenta development 4.11
regulation of phosphatidylinositol 3−kinase signaling 4.08
cAMP metabolic process 4.08
cyclic purine nucleotide metabolic process 4.07
regulation of STAT cascade 4.06
regulation of JAK−STAT cascade 4.06
cyclic nucleotide biosynthetic process 4.06
regulation of cyclic nucleotide metabolic process 4.02
skeletal muscle tissue development 4.01
endothelial cell migration 4.01
phosphatidylinositol 3−kinase signaling 3.96
skeletal muscle organ development 3.95
glycosaminoglycan metabolic process 3.95
STAT cascade 3.93
JAK−STAT cascade 3.93
establishment or maintenance of cell polarity 3.93
regulation of epithelial cell migration 3.92
positive regulation of mitochondrion organization 3.92
aminoglycan metabolic process 3.9
protein kinase regulator activity 3.89
carbohydrate derivative catabolic process 3.86
cyclic nucleotide metabolic process 3.82
actin polymerization or depolymerization 3.8
kinase regulator activity 3.78
response to mechanical stimulus 3.77
regulation of ion homeostasis 3.77
regulation of purine nucleotide metabolic process 3.74
phosphatidylinositol−mediated signaling 3.74
sulfur compound biosynthetic process 3.73
inositol lipid−mediated signaling 3.73
regulation of nucleotide metabolic process 3.71
negative regulation of cell migration 3.7
glycosaminoglycan binding 3.69
regulation of angiogenesis 3.69
cell cortex 3.65
regulation of mitochondrion organization 3.64
negative regulation of cell motility 3.64
epithelial cell migration 3.63
regulation of cellular response to growth factor stimulus 3.62
epithelium migration 3.62
negative regulation of protein kinase activity 3.61
tissue migration 3.59
regulation of vasculature development 3.59
purine ribonucleotide biosynthetic process 3.58
macroautophagy 3.58
purine nucleotide biosynthetic process 3.57
negative regulation of kinase activity 3.54
ribonucleotide biosynthetic process 3.53
purine−containing compound biosynthetic process 3.53
negative regulation of locomotion 3.52
negative regulation of cellular component movement 3.52
ribose phosphate biosynthetic process 3.51
peptidyl−serine modification 3.48
regulation of autophagy 3.47
kidney development 3.47
cytoplasmic region 3.46
aging 3.45
renal system development 3.42
response to lipopolysaccharide 3.34
nucleotide biosynthetic process 3.34
nucleoside phosphate biosynthetic process 3.33
response to molecule of bacterial origin 3.3
urogenital system development 3.29
ameboidal−type cell migration 3.26
actin filament organization 3.26
striated muscle tissue development 3.24
extracellular structure organization 3.24
extracellular matrix organization 3.24
proteinaceous extracellular matrix 3.24
negative regulation of transferase activity 3.21
regulation of membrane potential 3.2
muscle tissue development 3.2
muscle organ development 3.2
glycoprotein biosynthetic process 3.2
organonitrogen compound catabolic process 3.18
sulfur compound metabolic process 3.17
negative regulation of protein phosphorylation 3.1
enzyme inhibitor activity 3.09
glycoprotein metabolic process 3.08
actin binding 3.05
reproductive structure development 3.03
reproductive system development 3.02
angiogenesis 3.02
negative regulation of phosphorylation 3.01
actin cytoskeleton 3
autophagy 2.98
regulation of homeostatic process 2.97
negative regulation of intracellular signal transduction 2.94
blood vessel morphogenesis 2.85
purine ribonucleotide metabolic process 2.84
GO Terms
CC
BP
MF

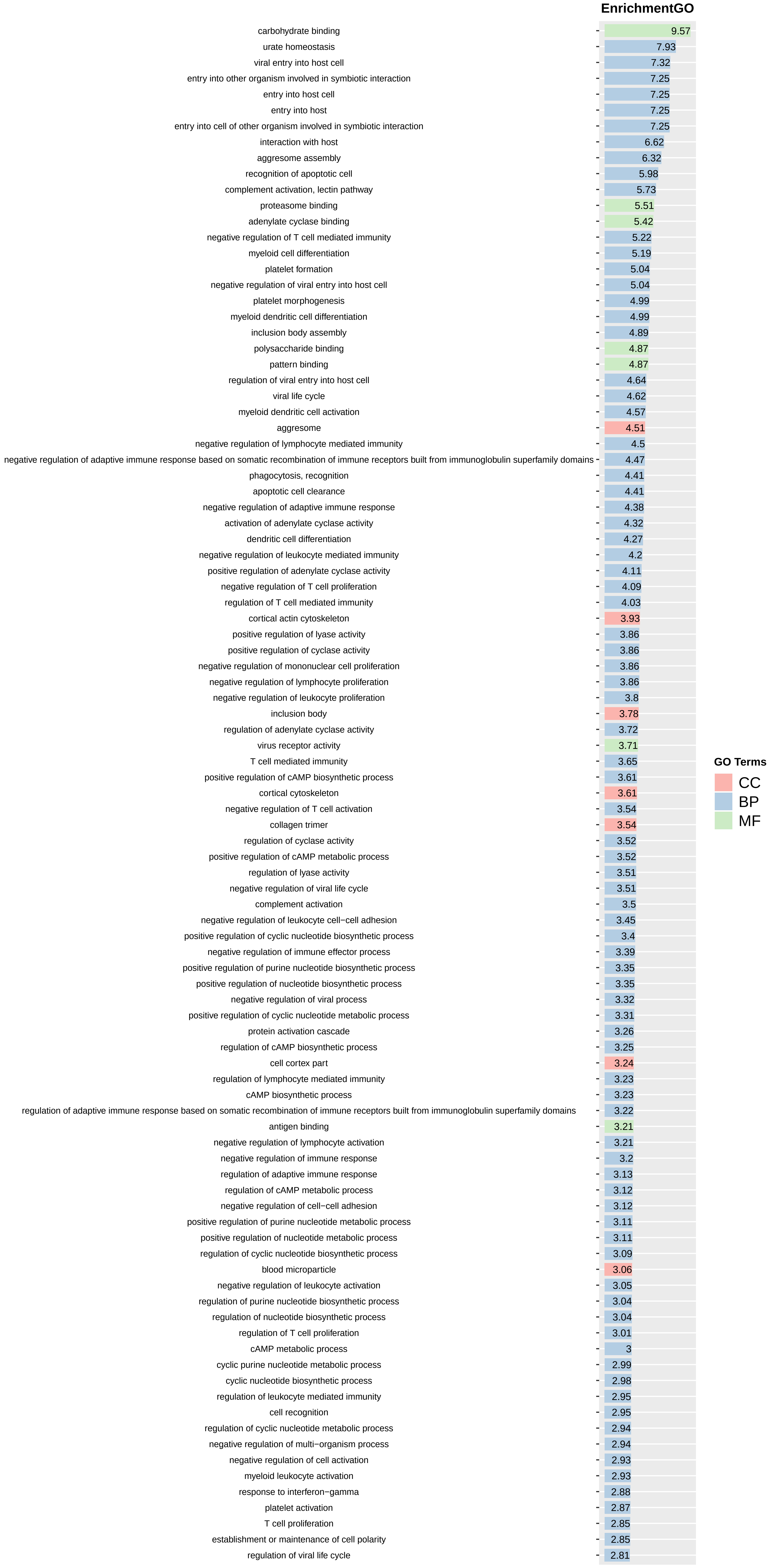
EnrichmentGO
carbohydrate binding
9.57
urate homeostasis
7.93
viral entry into host cell
7.32
entry into other organism involved in symbiotic interaction
7.25
entry into host cell
7.25
entry into host
7.25
entry into cell of other organism involved in symbiotic interaction
7.25
interaction with host
6.62
aggresome assembly
6.32
recognition of apoptotic cell
5.98
complement activation, lectin pathway
5.73
proteasome binding
5.51
adenylate cyclase binding
5.42
negative regulation of T cell mediated immunity
5.22
myeloid cell differentiation
5.19
platelet formation
5.04
negative regulation of viral entry into host cell
5.04
platelet morphogenesis
4.99
myeloid dendritic cell differentiation
4.99
inclusion body assembly
4.89
polysaccharide binding
4.87
pattern binding
4.87
regulation of viral entry into host cell
4.64
viral life cycle
4.62
myeloid dendritic cell activation
4.57
aggresome
4.51
negative regulation of lymphocyte mediated immunity
4.5
negative regulation of adaptive immune response based on somatic recombination of immune receptors built from immunoglobulin superfamily domains
4.47
phagocytosis, recognition
4.41
apoptotic cell clearance
4.41
negative regulation of adaptive immune response
4.38
activation of adenylate cyclase activity
4.32
dendritic cell differentiation
4.27
negative regulation of leukocyte mediated immunity
4.2
positive regulation of adenylate cyclase activity
4.11
negative regulation of T cell proliferation
4.09
regulation of T cell mediated immunity
4.03
cortical actin cytoskeleton
3.93
positive regulation of lyase activity
3.86
positive regulation of cyclase activity
3.86
negative regulation of mononuclear cell proliferation
3.86
negative regulation of lymphocyte proliferation
3.86
negative regulation of leukocyte proliferation
3.8
inclusion body
3.78
regulation of adenylate cyclase activity
3.72
virus receptor activity
3.71
T cell mediated immunity
3.65
positive regulation of cAMP biosynthetic process
3.61
cortical cytoskeleton
3.61
negative regulation of T cell activation
3.54
collagen trimer
3.54
regulation of cyclase activity
3.52
positive regulation of cAMP metabolic process
3.52
regulation of lyase activity
3.51
negative regulation of viral life cycle
3.51
complement activation
3.5
negative regulation of leukocyte cell−cell adhesion
3.45
positive regulation of cyclic nucleotide biosynthetic process
3.4
negative regulation of immune effector process
3.39
positive regulation of purine nucleotide biosynthetic process
3.35
positive regulation of nucleotide biosynthetic process
3.35
negative regulation of viral process
3.32
positive regulation of cyclic nucleotide metabolic process
3.31
protein activation cascade
3.26
regulation of cAMP biosynthetic process
3.25
cell cortex part
3.24
regulation of lymphocyte mediated immunity
3.23
cAMP biosynthetic process
3.23
regulation of adaptive immune response based on somatic recombination of immune receptors built from immunoglobulin superfamily domains
3.22
antigen binding
3.21
negative regulation of lymphocyte activation
3.21
negative regulation of immune response
3.2
regulation of adaptive immune response
3.13
regulation of cAMP metabolic process
3.12
negative regulation of cell−cell adhesion
3.12
positive regulation of purine nucleotide metabolic process
3.11
positive regulation of nucleotide metabolic process
3.11
regulation of cyclic nucleotide biosynthetic process
3.09
blood microparticle
3.06
negative regulation of leukocyte activation
3.05
regulation of purine nucleotide biosynthetic process
3.04
regulation of nucleotide biosynthetic process
3.04
regulation of T cell proliferation
3.01
cAMP metabolic process
3
cyclic purine nucleotide metabolic process
2.99
cyclic nucleotide biosynthetic process
2.98
regulation of leukocyte mediated immunity
2.95
cell recognition
2.95
regulation of cyclic nucleotide metabolic process
2.94
negative regulation of multi−organism process
2.94
negative regulation of cell activation
2.93
myeloid leukocyte activation
2.93
response to interferon−gamma
2.88
platelet activation
2.87
T cell proliferation
2.85
establishment or maintenance of cell polarity
2.85
regulation of viral life cycle
2.81
GO Terms
CC
BP
MF

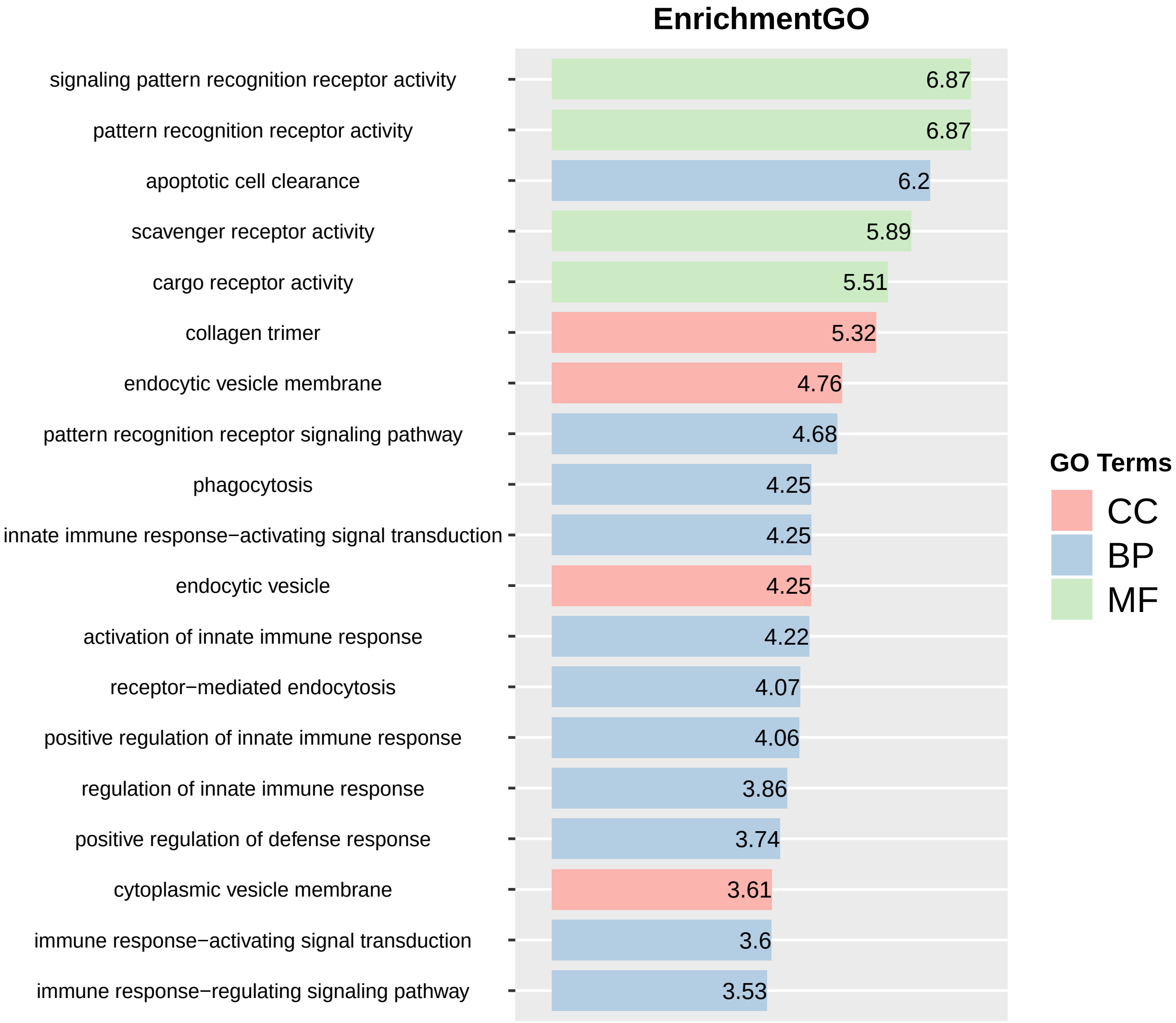
EnrichmentGO
signaling pattern recognition receptor activity
pattern recognition receptor activity
apoptotic cell clearance
scavenger receptor activity
cargo receptor activity
collagen trimer
endocytic vesicle membrane
pattern recognition receptor signaling pathway
phagocytosis
innate immune response−activating signal transduction
endocytic vesicle
activation of innate immune response
receptor−mediated endocytosis
positive regulation of innate immune response
regulation of innate immune response
positive regulation of defense response
cytoplasmic vesicle membrane
immune response−activating signal transduction
immune response−regulating signaling pathway
6.87
6.87
6.2
5.89
5.51
5.32
4.76
4.68
4.25
4.25
4.25
4.22
4.07
4.06
3.86
3.74
3.61
3.6
3.53
GO Terms
CC
BP
MF

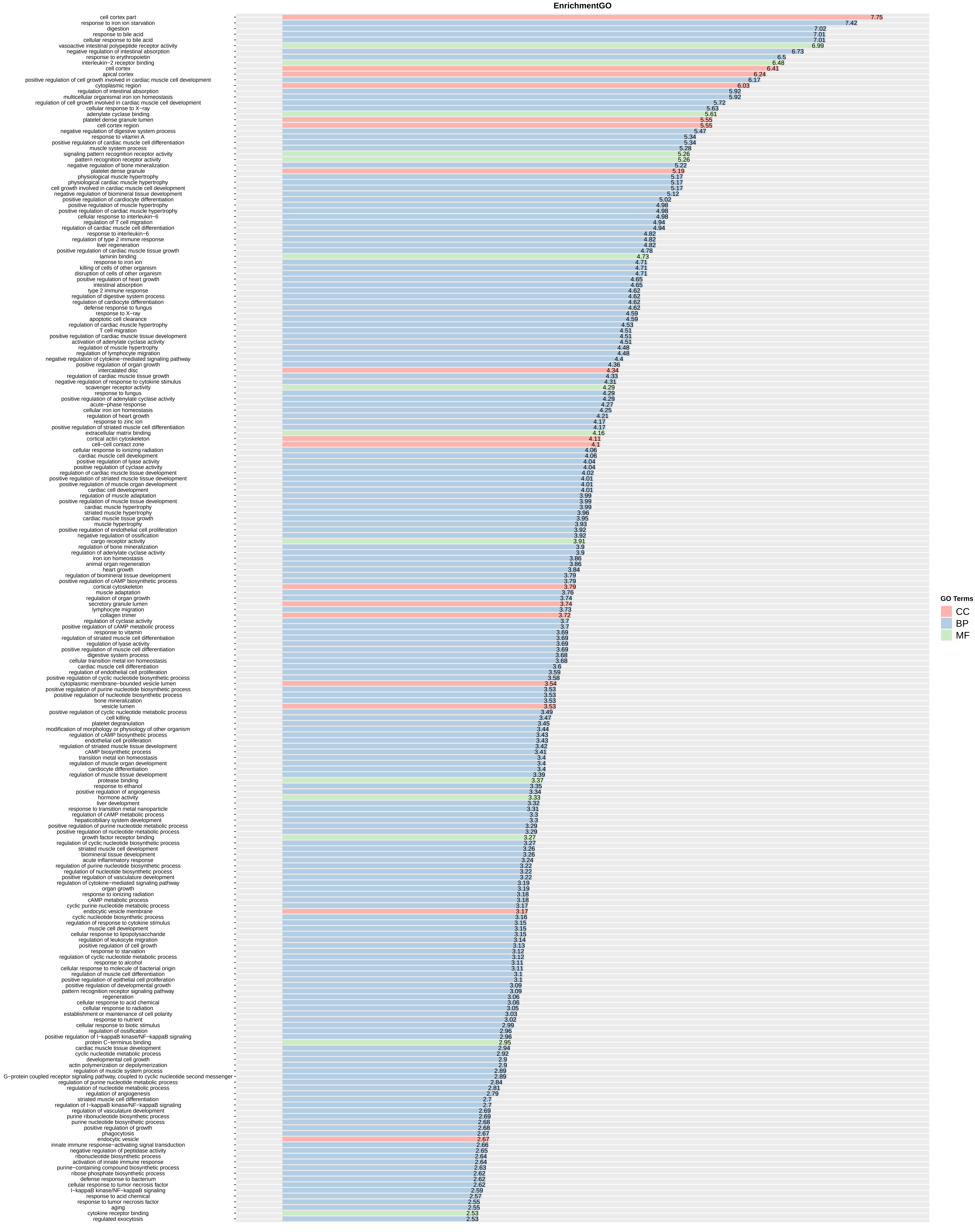

EnrichmentGO
GO Terms
CC
BP
MF
cell cortex part 7.75
response to iron ion starvation 7.42
digestion 7.02
response to bile acid 7.01
cellular response to bile acid 7.01
vasoactive intestinal polypeptide receptor activity 6.99
negative regulation of intestinal absorption 6.73
response to erythropoietin 6.5
interleukin−2 receptor binding 6.48
cell cortex 6.41
apical cortex 6.24
positive regulation of cell growth involved in cardiac muscle cell development 6.17
cytoplasmic region 6.03
regulation of intestinal absorption 5.92
multicellular organismal iron ion homeostasis 5.92
regulation of cell growth involved in cardiac muscle cell development 5.72
cellular response to X−ray 5.63
adenylate cyclase binding 5.61
platelet dense granule lumen 5.55
cell cortex region 5.55
negative regulation of digestive system process 5.47
response to vitamin A 5.34
positive regulation of cardiac muscle cell differentiation 5.34
muscle system process 5.28
signaling pattern recognition receptor activity 5.26
pattern recognition receptor activity 5.26
negative regulation of bone mineralization 5.22
platelet dense granule 5.19
physiological muscle hypertrophy 5.17
physiological cardiac muscle hypertrophy 5.17
cell growth involved in cardiac muscle cell development 5.17
negative regulation of biomineral tissue development 5.12
positive regulation of cardiocyte differentiation 5.02
positive regulation of muscle hypertrophy 4.98
positive regulation of cardiac muscle hypertrophy 4.98
cellular response to interleukin−6 4.98
regulation of T cell migration 4.94
regulation of cardiac muscle cell differentiation 4.94
response to interleukin−6 4.82
regulation of type 2 immune response 4.82
liver regeneration 4.82
positive regulation of cardiac muscle tissue growth 4.78
laminin binding 4.73
response to iron ion 4.71
killing of cells of other organism 4.71
disruption of cells of other organism 4.71
positive regulation of heart growth 4.65
intestinal absorption 4.65
type 2 immune response 4.62
regulation of digestive system process 4.62
regulation of cardiocyte differentiation 4.62
defense response to fungus 4.62
response to X−ray 4.59
apoptotic cell clearance 4.59
regulation of cardiac muscle hypertrophy 4.53
T cell migration 4.51
positive regulation of cardiac muscle tissue development 4.51
activation of adenylate cyclase activity 4.51
regulation of muscle hypertrophy 4.48
regulation of lymphocyte migration 4.48
negative regulation of cytokine−mediated signaling pathway 4.4
positive regulation of organ growth 4.36
intercalated disc 4.34
regulation of cardiac muscle tissue growth 4.33
negative regulation of response to cytokine stimulus 4.31
scavenger receptor activity 4.29
response to fungus 4.29
positive regulation of adenylate cyclase activity 4.29
acute−phase response 4.27
cellular iron ion homeostasis 4.25
regulation of heart growth 4.21
response to zinc ion 4.17
positive regulation of striated muscle cell differentiation 4.17
extracellular matrix binding 4.16
cortical actin cytoskeleton 4.11
cell−cell contact zone 4.1
cellular response to ionizing radiation 4.06
cardiac muscle cell development 4.06
positive regulation of lyase activity 4.04
positive regulation of cyclase activity 4.04
regulation of cardiac muscle tissue development 4.02
positive regulation of striated muscle tissue development 4.01
positive regulation of muscle organ development 4.01
cardiac cell development 4.01
regulation of muscle adaptation 3.99
positive regulation of muscle tissue development 3.99
cardiac muscle hypertrophy 3.99
striated muscle hypertrophy 3.96
cardiac muscle tissue growth 3.95
muscle hypertrophy 3.93
positive regulation of endothelial cell proliferation 3.92
negative regulation of ossification 3.92
cargo receptor activity 3.91
regulation of bone mineralization 3.9
regulation of adenylate cyclase activity 3.9
iron ion homeostasis 3.86
animal organ regeneration 3.86
heart growth 3.84
regulation of biomineral tissue development 3.79
positive regulation of cAMP biosynthetic process 3.79
cortical cytoskeleton 3.79
muscle adaptation 3.76
regulation of organ growth 3.74
secretory granule lumen 3.74
lymphocyte migration 3.73
collagen trimer 3.72
regulation of cyclase activity 3.7
positive regulation of cAMP metabolic process 3.7
response to vitamin 3.69
regulation of striated muscle cell differentiation 3.69
regulation of lyase activity 3.69
positive regulation of muscle cell differentiation 3.69
digestive system process 3.68
cellular transition metal ion homeostasis 3.68
cardiac muscle cell differentiation 3.6
regulation of endothelial cell proliferation 3.59
positive regulation of cyclic nucleotide biosynthetic process 3.58
cytoplasmic membrane−bounded vesicle lumen 3.54
positive regulation of purine nucleotide biosynthetic process 3.53
positive regulation of nucleotide biosynthetic process 3.53
bone mineralization 3.53
vesicle lumen 3.53
positive regulation of cyclic nucleotide metabolic process 3.49
cell killing 3.47
platelet degranulation 3.45
modification of morphology or physiology of other organism 3.44
regulation of cAMP biosynthetic process 3.43
endothelial cell proliferation 3.43
regulation of striated muscle tissue development 3.42
cAMP biosynthetic process 3.41
transition metal ion homeostasis 3.4
regulation of muscle organ development 3.4
cardiocyte differentiation 3.4
regulation of muscle tissue development 3.39
protease binding 3.37
response to ethanol 3.35
positive regulation of angiogenesis 3.34
hormone activity 3.33
liver development 3.32
response to transition metal nanoparticle 3.31
regulation of cAMP metabolic process 3.3
hepaticobiliary system development 3.3
positive regulation of purine nucleotide metabolic process 3.29
positive regulation of nucleotide metabolic process 3.29
growth factor receptor binding 3.27
regulation of cyclic nucleotide biosynthetic process 3.27
striated muscle cell development 3.26
biomineral tissue development 3.26
acute inflammatory response 3.24
regulation of purine nucleotide biosynthetic process 3.22
regulation of nucleotide biosynthetic process 3.22
positive regulation of vasculature development 3.22
regulation of cytokine−mediated signaling pathway 3.19
organ growth 3.19
response to ionizing radiation 3.18
cAMP metabolic process 3.18
cyclic purine nucleotide metabolic process 3.17
endocytic vesicle membrane 3.17
cyclic nucleotide biosynthetic process 3.16
regulation of response to cytokine stimulus 3.15
muscle cell development 3.15
cellular response to lipopolysaccharide 3.15
regulation of leukocyte migration 3.14
positive regulation of cell growth 3.13
response to starvation 3.12
regulation of cyclic nucleotide metabolic process 3.12
response to alcohol 3.11
cellular response to molecule of bacterial origin 3.11
regulation of muscle cell differentiation 3.1
positive regulation of epithelial cell proliferation 3.1
positive regulation of developmental growth 3.09
pattern recognition receptor signaling pathway 3.09
regeneration 3.06
cellular response to acid chemical 3.06
cellular response to radiation 3.05
establishment or maintenance of cell polarity 3.03
response to nutrient 3.02
cellular response to biotic stimulus 2.99
regulation of ossification 2.96
positive regulation of I−kappaB kinase/NF−kappaB signaling 2.96
protein C−terminus binding 2.95
cardiac muscle tissue development 2.94
cyclic nucleotide metabolic process 2.92
developmental cell growth 2.9
actin polymerization or depolymerization 2.9
regulation of muscle system process 2.89
G−protein coupled receptor signaling pathway, coupled to cyclic nucleotide second messenger 2.89
regulation of purine nucleotide metabolic process 2.84
regulation of nucleotide metabolic process 2.81
regulation of angiogenesis 2.79
striated muscle cell differentiation 2.7
regulation of I−kappaB kinase/NF−kappaB signaling 2.7
regulation of vasculature development 2.69
purine ribonucleotide biosynthetic process 2.69
purine nucleotide biosynthetic process 2.68
positive regulation of growth 2.68
phagocytosis 2.67
endocytic vesicle 2.67
innate immune response−activating signal transduction 2.66
negative regulation of peptidase activity 2.65
ribonucleotide biosynthetic process 2.64
activation of innate immune response 2.64
purine−containing compound biosynthetic process 2.63
ribose phosphate biosynthetic process 2.62
defense response to bacterium 2.62
cellular response to tumor necrosis factor 2.62
I−kappaB kinase/NF−kappaB signaling 2.59
response to acid chemical 2.57
response to tumor necrosis factor 2.55
aging 2.55
cytokine receptor binding 2.53
regulated exocytosis 2.53

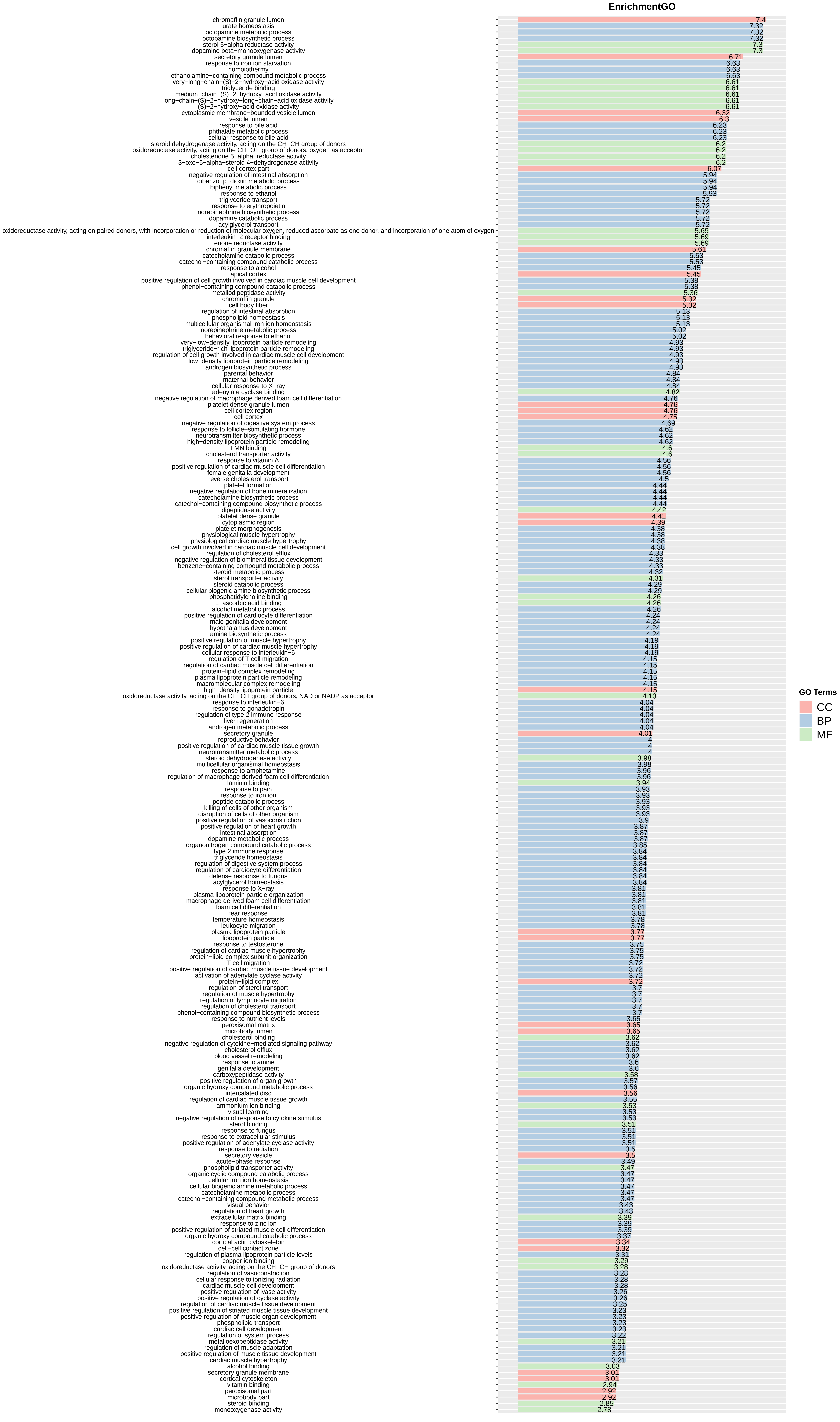
EnrichmentGO
chromaffin granule lumen 7.4
urate homeostasis 7.32
octopamine metabolic process 7.32
octopamine biosynthetic process 7.32
sterol 5−alpha reductase activity 7.3
dopamine beta−monooxygenase activity 7.3
secretory granule lumen 6.71
response to iron ion starvation 6.63
homoiothermy 6.63
ethanolamine−containing compound metabolic process 6.63
very−long−chain−(S)−2−hydroxy−acid oxidase activity 6.61
triglyceride binding 6.61
medium−chain−(S)−2−hydroxy−acid oxidase activity 6.61
long−chain−(S)−2−hydroxy−long−chain−acid oxidase activity 6.61
(S)−2−hydroxy−acid oxidase activity 6.61
cytoplasmic membrane−bounded vesicle lumen 6.32
vesicle lumen 6.3
response to bile acid 6.23
phthalate metabolic process 6.23
cellular response to bile acid 6.23
steroid dehydrogenase activity, acting on the CH−CH group of donors 6.2
oxidoreductase activity, acting on the CH−OH group of donors, oxygen as acceptor 6.2
cholestenone 5−alpha−reductase activity 6.2
3−oxo−5−alpha−steroid 4−dehydrogenase activity 6.2
cell cortex part 6.07
negative regulation of intestinal absorption 5.94
dibenzo−p−dioxin metabolic process 5.94
biphenyl metabolic process 5.94
response to ethanol 5.93
triglyceride transport 5.72
response to erythropoietin 5.72
norepinephrine biosynthetic process 5.72
dopamine catabolic process 5.72
acylglycerol transport 5.72
oxidoreductase activity, acting on paired donors, with incorporation or reduction of molecular oxygen, reduced ascorbate as one donor, and incorporation of one atom of oxygen 5.69
interleukin−2 receptor binding 5.69
enone reductase activity 5.69
chromaffin granule membrane 5.61
catecholamine catabolic process 5.53
catechol−containing compound catabolic process 5.53
response to alcohol 5.45
apical cortex 5.45
positive regulation of cell growth involved in cardiac muscle cell development 5.38
phenol−containing compound catabolic process 5.38
metallodipeptidase activity 5.36
chromaffin granule 5.32
cell body fiber 5.32
regulation of intestinal absorption 5.13
phospholipid homeostasis 5.13
multicellular organismal iron ion homeostasis 5.13
norepinephrine metabolic process 5.02
behavioral response to ethanol 5.02
very−low−density lipoprotein particle remodeling 4.93
triglyceride−rich lipoprotein particle remodeling 4.93
regulation of cell growth involved in cardiac muscle cell development 4.93
low−density lipoprotein particle remodeling 4.93
androgen biosynthetic process 4.93
parental behavior 4.84
maternal behavior 4.84
cellular response to X−ray 4.84
adenylate cyclase binding 4.82
negative regulation of macrophage derived foam cell differentiation 4.76
platelet dense granule lumen 4.76
cell cortex region 4.76
cell cortex 4.75
negative regulation of digestive system process 4.69
response to follicle−stimulating hormone 4.62
neurotransmitter biosynthetic process 4.62
high−density lipoprotein particle remodeling 4.62
FMN binding 4.6
cholesterol transporter activity 4.6
response to vitamin A 4.56
positive regulation of cardiac muscle cell differentiation 4.56
female genitalia development 4.56
reverse cholesterol transport 4.5
platelet formation 4.44
negative regulation of bone mineralization 4.44
catecholamine biosynthetic process 4.44
catechol−containing compound biosynthetic process 4.44
dipeptidase activity 4.42
platelet dense granule 4.41
cytoplasmic region 4.39
platelet morphogenesis 4.38
physiological muscle hypertrophy 4.38
physiological cardiac muscle hypertrophy 4.38
cell growth involved in cardiac muscle cell development 4.38
regulation of cholesterol efflux 4.33
negative regulation of biomineral tissue development 4.33
benzene−containing compound metabolic process 4.33
steroid metabolic process 4.32
sterol transporter activity 4.31
steroid catabolic process 4.29
cellular biogenic amine biosynthetic process 4.29
phosphatidylcholine binding 4.26
L−ascorbic acid binding 4.26
alcohol metabolic process 4.26
positive regulation of cardiocyte differentiation 4.24
male genitalia development 4.24
hypothalamus development 4.24
amine biosynthetic process 4.24
positive regulation of muscle hypertrophy 4.19
positive regulation of cardiac muscle hypertrophy 4.19
cellular response to interleukin−6 4.19
regulation of T cell migration 4.15
regulation of cardiac muscle cell differentiation 4.15
protein−lipid complex remodeling 4.15
plasma lipoprotein particle remodeling 4.15
macromolecular complex remodeling 4.15
high−density lipoprotein particle 4.15
oxidoreductase activity, acting on the CH−CH group of donors, NAD or NADP as acceptor 4.13
response to interleukin−6 4.04
response to gonadotropin 4.04
regulation of type 2 immune response 4.04
liver regeneration 4.04
androgen metabolic process 4.04
secretory granule 4.01
reproductive behavior 4
positive regulation of cardiac muscle tissue growth 4
neurotransmitter metabolic process 4
steroid dehydrogenase activity 3.98
multicellular organismal homeostasis 3.98
response to amphetamine 3.96
regulation of macrophage derived foam cell differentiation 3.96
laminin binding 3.94
response to pain 3.93
response to iron ion 3.93
peptide catabolic process 3.93
killing of cells of other organism 3.93
disruption of cells of other organism 3.93
positive regulation of vasoconstriction 3.9
positive regulation of heart growth 3.87
intestinal absorption 3.87
dopamine metabolic process 3.87
organonitrogen compound catabolic process 3.85
type 2 immune response 3.84
triglyceride homeostasis 3.84
regulation of digestive system process 3.84
regulation of cardiocyte differentiation 3.84
defense response to fungus 3.84
acylglycerol homeostasis 3.84
response to X−ray 3.81
plasma lipoprotein particle organization 3.81
macrophage derived foam cell differentiation 3.81
foam cell differentiation 3.81
fear response 3.81
temperature homeostasis 3.78
leukocyte migration 3.78
plasma lipoprotein particle 3.77
lipoprotein particle 3.77
response to testosterone 3.75
regulation of cardiac muscle hypertrophy 3.75
protein−lipid complex subunit organization 3.75
T cell migration 3.72
positive regulation of cardiac muscle tissue development 3.72
activation of adenylate cyclase activity 3.72
protein−lipid complex 3.72
regulation of sterol transport 3.7
regulation of muscle hypertrophy 3.7
regulation of lymphocyte migration 3.7
regulation of cholesterol transport 3.7
phenol−containing compound biosynthetic process 3.7
response to nutrient levels 3.65
peroxisomal matrix 3.65
microbody lumen 3.65
cholesterol binding 3.62
negative regulation of cytokine−mediated signaling pathway 3.62
cholesterol efflux 3.62
blood vessel remodeling 3.62
response to amine 3.6
genitalia development 3.6
carboxypeptidase activity 3.58
positive regulation of organ growth 3.57
organic hydroxy compound metabolic process 3.56
intercalated disc 3.56
regulation of cardiac muscle tissue growth 3.55
ammonium ion binding 3.53
visual learning 3.53
negative regulation of response to cytokine stimulus 3.53
sterol binding 3.51
response to fungus 3.51
response to extracellular stimulus 3.51
positive regulation of adenylate cyclase activity 3.51
response to radiation 3.5
secretory vesicle 3.5
acute−phase response 3.49
phospholipid transporter activity 3.47
organic cyclic compound catabolic process 3.47
cellular iron ion homeostasis 3.47
cellular biogenic amine metabolic process 3.47
catecholamine metabolic process 3.47
catechol−containing compound metabolic process 3.47
visual behavior 3.43
regulation of heart growth 3.43
extracellular matrix binding 3.39
response to zinc ion 3.39
positive regulation of striated muscle cell differentiation 3.39
organic hydroxy compound catabolic process 3.37
cortical actin cytoskeleton 3.34
cell−cell contact zone 3.32
regulation of plasma lipoprotein particle levels 3.31
copper ion binding 3.29
oxidoreductase activity, acting on the CH−CH group of donors 3.28
regulation of vasoconstriction 3.28
cellular response to ionizing radiation 3.28
cardiac muscle cell development 3.28
positive regulation of lyase activity 3.26
positive regulation of cyclase activity 3.26
regulation of cardiac muscle tissue development 3.25
positive regulation of striated muscle tissue development 3.23
positive regulation of muscle organ development 3.23
phospholipid transport 3.23
cardiac cell development 3.23
regulation of system process 3.22
metalloexopeptidase activity 3.21
regulation of muscle adaptation 3.21
positive regulation of muscle tissue development 3.21
cardiac muscle hypertrophy 3.21
alcohol binding 3.03
secretory granule membrane 3.01
cortical cytoskeleton 3.01
vitamin binding 2.94
peroxisomal part 2.92
microbody part 2.92
steroid binding 2.85
monooxygenase activity 2.78
GO Terms
CC
BP
MF

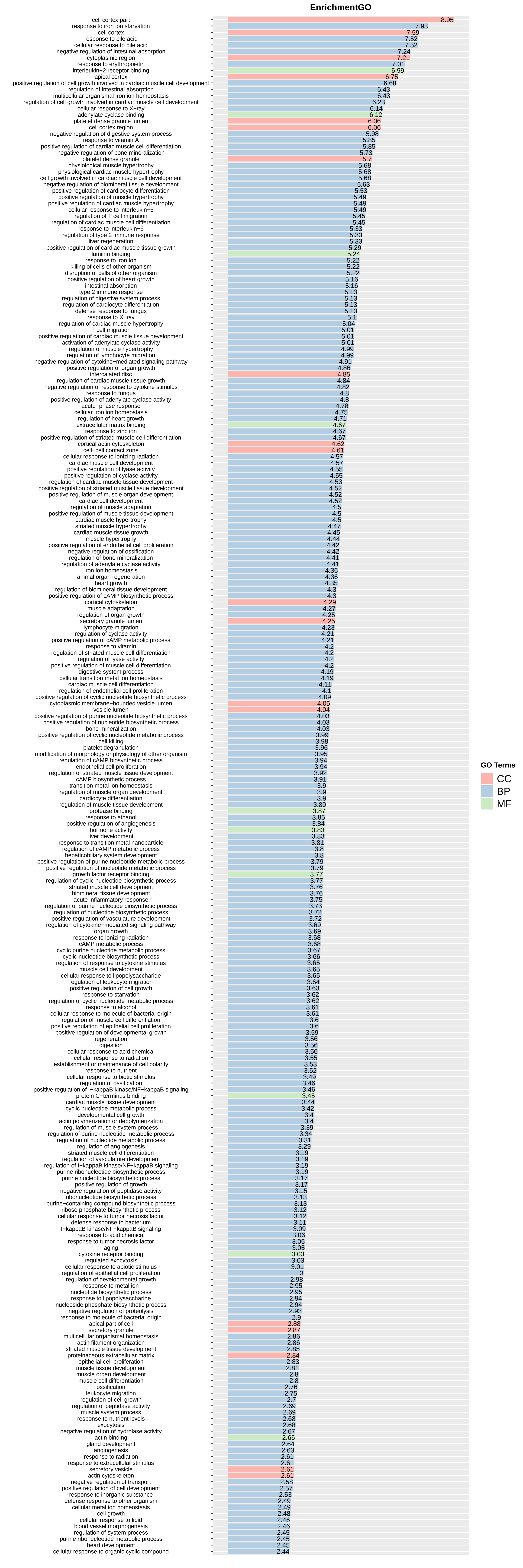
EnrichmentGO
cell cortex part 8.95
response to iron ion starvation 7.93
cell cortex 7.59
response to bile acid 7.52
cellular response to bile acid 7.52
negative regulation of intestinal absorption 7.24
cytoplasmic region 7.21
response to erythropoietin 7.01
interleukin−2 receptor binding 6.99
apical cortex 6.75
positive regulation of cell growth involved in cardiac muscle cell development 6.68
regulation of intestinal absorption 6.43
multicellular organismal iron ion homeostasis 6.43
regulation of cell growth involved in cardiac muscle cell development 6.23
cellular response to X−ray 6.14
adenylate cyclase binding 6.12
platelet dense granule lumen 6.06
cell cortex region 6.06
negative regulation of digestive system process 5.98
response to vitamin A 5.85
positive regulation of cardiac muscle cell differentiation 5.85
negative regulation of bone mineralization 5.73
platelet dense granule 5.7
physiological muscle hypertrophy 5.68
physiological cardiac muscle hypertrophy 5.68
cell growth involved in cardiac muscle cell development 5.68
negative regulation of biomineral tissue development 5.63
positive regulation of cardiocyte differentiation 5.53
positive regulation of muscle hypertrophy 5.49
positive regulation of cardiac muscle hypertrophy 5.49
cellular response to interleukin−6 5.49
regulation of T cell migration 5.45
regulation of cardiac muscle cell differentiation 5.45
response to interleukin−6 5.33
regulation of type 2 immune response 5.33
liver regeneration 5.33
positive regulation of cardiac muscle tissue growth 5.29
laminin binding 5.24
response to iron ion 5.22
killing of cells of other organism 5.22
disruption of cells of other organism 5.22
positive regulation of heart growth 5.16
intestinal absorption 5.16
type 2 immune response 5.13
regulation of digestive system process 5.13
regulation of cardiocyte differentiation 5.13
defense response to fungus 5.13
response to X−ray 5.1
regulation of cardiac muscle hypertrophy 5.04
T cell migration 5.01
positive regulation of cardiac muscle tissue development 5.01
activation of adenylate cyclase activity 5.01
regulation of muscle hypertrophy 4.99
regulation of lymphocyte migration 4.99
negative regulation of cytokine−mediated signaling pathway 4.91
positive regulation of organ growth 4.86
intercalated disc 4.85
regulation of cardiac muscle tissue growth 4.84
negative regulation of response to cytokine stimulus 4.82
response to fungus 4.8
positive regulation of adenylate cyclase activity 4.8
acute−phase response 4.78
cellular iron ion homeostasis 4.75
regulation of heart growth 4.71
extracellular matrix binding 4.67
response to zinc ion 4.67
positive regulation of striated muscle cell differentiation 4.67
cortical actin cytoskeleton 4.62
cell−cell contact zone 4.61
cellular response to ionizing radiation 4.57
cardiac muscle cell development 4.57
positive regulation of lyase activity 4.55
positive regulation of cyclase activity 4.55
regulation of cardiac muscle tissue development 4.53
positive regulation of striated muscle tissue development 4.52
positive regulation of muscle organ development 4.52
cardiac cell development 4.52
regulation of muscle adaptation 4.5
positive regulation of muscle tissue development 4.5
cardiac muscle hypertrophy 4.5
striated muscle hypertrophy 4.47
cardiac muscle tissue growth 4.45
muscle hypertrophy 4.44
positive regulation of endothelial cell proliferation 4.42
negative regulation of ossification 4.42
regulation of bone mineralization 4.41
regulation of adenylate cyclase activity 4.41
iron ion homeostasis 4.36
animal organ regeneration 4.36
heart growth 4.35
regulation of biomineral tissue development 4.3
positive regulation of cAMP biosynthetic process 4.3
cortical cytoskeleton 4.29
muscle adaptation 4.27
regulation of organ growth 4.25
secretory granule lumen 4.25
lymphocyte migration 4.23
regulation of cyclase activity 4.21
positive regulation of cAMP metabolic process 4.21
response to vitamin 4.2
regulation of striated muscle cell differentiation 4.2
regulation of lyase activity 4.2
positive regulation of muscle cell differentiation 4.2
digestive system process 4.19
cellular transition metal ion homeostasis 4.19
cardiac muscle cell differentiation 4.11
regulation of endothelial cell proliferation 4.1
positive regulation of cyclic nucleotide biosynthetic process 4.09
cytoplasmic membrane−bounded vesicle lumen 4.05
vesicle lumen 4.04
positive regulation of purine nucleotide biosynthetic process 4.03
positive regulation of nucleotide biosynthetic process 4.03
bone mineralization 4.03
positive regulation of cyclic nucleotide metabolic process 3.99
cell killing 3.98
platelet degranulation 3.96
modification of morphology or physiology of other organism 3.95
regulation of cAMP biosynthetic process 3.94
endothelial cell proliferation 3.94
regulation of striated muscle tissue development 3.92
cAMP biosynthetic process 3.91
transition metal ion homeostasis 3.9
regulation of muscle organ development 3.9
cardiocyte differentiation 3.9
regulation of muscle tissue development 3.89
protease binding 3.87
response to ethanol 3.85
positive regulation of angiogenesis 3.84
hormone activity 3.83
liver development 3.83
response to transition metal nanoparticle 3.81
regulation of cAMP metabolic process 3.8
hepaticobiliary system development 3.8
positive regulation of purine nucleotide metabolic process 3.79
positive regulation of nucleotide metabolic process 3.79
growth factor receptor binding 3.77
regulation of cyclic nucleotide biosynthetic process 3.77
striated muscle cell development 3.76
biomineral tissue development 3.76
acute inflammatory response 3.75
regulation of purine nucleotide biosynthetic process 3.73
regulation of nucleotide biosynthetic process 3.72
positive regulation of vasculature development 3.72
regulation of cytokine−mediated signaling pathway 3.69
organ growth 3.69
response to ionizing radiation 3.68
cAMP metabolic process 3.68
cyclic purine nucleotide metabolic process 3.67
cyclic nucleotide biosynthetic process 3.66
regulation of response to cytokine stimulus 3.65
muscle cell development 3.65
cellular response to lipopolysaccharide 3.65
regulation of leukocyte migration 3.64
positive regulation of cell growth 3.63
response to starvation 3.62
regulation of cyclic nucleotide metabolic process 3.62
response to alcohol 3.61
cellular response to molecule of bacterial origin 3.61
regulation of muscle cell differentiation 3.6
positive regulation of epithelial cell proliferation 3.6
positive regulation of developmental growth 3.59
regeneration 3.56
digestion 3.56
cellular response to acid chemical 3.56
cellular response to radiation 3.55
establishment or maintenance of cell polarity 3.53
response to nutrient 3.52
cellular response to biotic stimulus 3.49
regulation of ossification 3.46
positive regulation of I−kappaB kinase/NF−kappaB signaling 3.46
protein C−terminus binding 3.45
cardiac muscle tissue development 3.44
cyclic nucleotide metabolic process 3.42
developmental cell growth 3.4
actin polymerization or depolymerization 3.4
regulation of muscle system process 3.39
regulation of purine nucleotide metabolic process 3.34
regulation of nucleotide metabolic process 3.31
regulation of angiogenesis 3.29
striated muscle cell differentiation 3.19
regulation of vasculature development 3.19
regulation of I−kappaB kinase/NF−kappaB signaling 3.19
purine ribonucleotide biosynthetic process 3.19
purine nucleotide biosynthetic process 3.17
positive regulation of growth 3.17
negative regulation of peptidase activity 3.15
ribonucleotide biosynthetic process 3.13
purine−containing compound biosynthetic process 3.13
ribose phosphate biosynthetic process 3.12
cellular response to tumor necrosis factor 3.12
defense response to bacterium 3.11
I−kappaB kinase/NF−kappaB signaling 3.09
response to acid chemical 3.06
response to tumor necrosis factor 3.05
aging 3.05
cytokine receptor binding 3.03
regulated exocytosis 3.03
cellular response to abiotic stimulus 3.01
regulation of epithelial cell proliferation 3
regulation of developmental growth 2.98
response to metal ion 2.95
nucleotide biosynthetic process 2.95
response to lipopolysaccharide 2.94
nucleoside phosphate biosynthetic process 2.94
negative regulation of proteolysis 2.93
response to molecule of bacterial origin 2.9
apical part of cell 2.88
secretory granule 2.87
multicellular organismal homeostasis 2.86
actin filament organization 2.86
striated muscle tissue development 2.85
proteinaceous extracellular matrix 2.84
epithelial cell proliferation 2.83
muscle tissue development 2.81
muscle organ development 2.8
muscle cell differentiation 2.8
ossification 2.76
leukocyte migration 2.75
regulation of cell growth 2.7
regulation of peptidase activity 2.69
muscle system process 2.69
response to nutrient levels 2.68
exocytosis 2.68
negative regulation of hydrolase activity 2.67
actin binding 2.66
gland development 2.64
angiogenesis 2.63
response to radiation 2.61
response to extracellular stimulus 2.61
secretory vesicle 2.61
actin cytoskeleton 2.61
negative regulation of transport 2.58
positive regulation of cell development 2.57
response to inorganic substance 2.53
defense response to other organism 2.49
cellular metal ion homeostasis 2.49
cell growth 2.48
cellular response to lipid 2.46
blood vessel morphogenesis 2.46
regulation of system process 2.45
purine ribonucleotide metabolic process 2.45
heart development 2.45
cellular response to organic cyclic compound 2.44
GO Terms
CC
BP
MF
