# Supplementary material for: Identification of key miRNAs in the progression of hepatocellular carcinoma using an integrated bioinformatics approach
Source: PeerJ. 2020 May 6;8:e9000. doi: 10.7717/peerj.9000 (PMC7210814; doi:10.7717/peerj.9000)
Supplement: Supplemental Information 3 [file peerj-08-9000-s003.pdf]

Table S1:
List of differentially expressed mRNAs

CYP1A2
MT1G
HAMP
MT1F
CYP3A4
FCN3
MT1M
MT1E
MT1X
CYP2C8
ADH4
APOF
CYP2E1
MT1A
TAT
CLEC1B
ADH1B
C9
FOS
HSD17B13
ADH1C
CYP2A6
HGFAC
AFM
LCAT
BBOX1
SLC22A1
CYP2C9
BHMT
SLCO1B3
PCK1
NAT2
GBA3
SLC10A1
IGFALS
NNMT
GHR
THRSP
CYP8B1
CLEC4G
DNASE1L3
HPD

F9
UGT2B10
CYP4A11
GCGR
MT2A
C6
PGLYRP2
LY6E
SDS
SPP2
FBP1
HSD11B1
CLRN3
LECT2
C8A
SLC27A5
PDK4
MT1H
FOSB
ANXA10
ALDOB
APOA5
SLC25A47
SERPINA11
SRD5A2
GLYAT
MBL2
KBTBD11
KLKB1
SHBG
BCHE
HBA2
ASPG
PLGLB1
CA2
C3P1
HSD17B6
HPX
HBB
RND3
ADH1A
AQP9
DBH
AOX1

KMO
PLG
EVC2
ASPDH
CDHR2
CPS1
ADIRF
C7
CYP4V2
CYP2A7
TCIM
CNDP1
SOCS2
HP
MT1IP
NPW
ENO3
TDO2
CYR61
G6PC
EGR1
FMO3
ADH6
CHST4
SNORD13
IGFBP1
TMEM27
SMIM24
HAO2
FTCD
AKR7A3
GSTA2
DCN
PON1
MAT1A
INMT
DUSP1
VIPR1
GNMT
CIDEB
FETUB
PHGDH
ANG
WDR72

RDH16
CYP2J2
IGFBP3
OTC
GATM
C8B
CMBL
ACSL1
SULT2A1
SLC27A2
AKR1D1
CFHR3
RIDA
PROZ
CYP39A1
ACACB
EPHX2
AGXT2
CAT
RCL1
GPT
DCXR
DPYS
CFI
HAO1
HRG
ALDH2
ST3GAL6
ACOT12
TTR
MARCO
DEFB1
PLIN2
ALDH6A1
PON3
ACAA1
CSRNP1
FXYD1
APCS
ALPL
AQP7P3
AGXT
NDRG2
OIT3

ETNPPL
ALDH8A1
OGDHL
CETP
PNPLA7
LEAP2
IDNK
F12
ALDH1L1
ACSM2A
C4BPA
ORM2
ZGPAT
GLS2
PZP
NOCT
ATOH8
SLC39A5
ASS1
UGT2B7
SLC38A4
A2M
SLC13A5
HSD17B2
ECM1
ARG1
GAGE6
SLC12A9
MCM5
SELENOM
HIST2H2AC
AURKB
RPS21
ZNF544
CDC45
RBM34
NPLOC4
GMFB
AGO2
DARS2
ACTN4
SGCE
ZNF581
CHKA

TESC
CLPTM1L
TUBA1B
CTNNA1
DNAJC9
HIST1H4C
RANGAP1
EEF1A2
HSPH1
RBM42
VPS37C
CHTOP
STT3A
PTPRF
UBE2C
NOL7
NABP2
UBE2Q2
B4GALT3
VAT1
SQSTM1
GTPBP4
KPNB1
NT5DC2
ZYX
CAPNS1
PDXK
RCC2
IMPDH2
H1F0
ZC3H3
WFS1
CKS2
ARL2
FBL
MTMR11
COL15A1
UCK2
GNPDA1
POLR2G
MFSD10
CREB3L2
TMCO3
TUBB2A

MPV17
SNX27
CS
SMARCC1
BLVRA
PIGU
TBCE
SPNS1
TK1
TRRAP
TOMM6
TMEM147
FAM20B
TRNP1
SEPT9
ATIC
NUP205
PA2G4
NCAPD2
PALLD
IRX3
SLC50A1
SF3A2
TPR
MARCKS
TMED3
POLR3C
FKBP11
PSMB4
NUP37
FAT1
LASP1
TUBB4B
RGCC
PARP12
PHPT1
HEATR1
COPA
TLCD1
PITX1
TUFT1
FAM50A
TUG1
EFTUD2

MCM3
DAP3
POGK
ZNF207
LSM2
GRAMD1A
ATP6V1F
NPC1
HSPA1A
PRPF3
DLK1
FBXL18
TXNRD1
LAGE3
HKDC1
CLDN15
FDPS
NUPR1
BAIAP2L1
HCFC1
CKAP2L
TMEM9
PYGB
GNPAT
MED20
SCAMP3
AP3B1
TUBA1A
H2AFZ
CLCN7
NDUFA4L2
AFP
NUSAP1
SERPINE2
NAXE
PLVAP
COL1A2
CFL1
ANXA2
RUSC1
CDK4
UQCC2
CPD
WASHC5

KIFC1
GMNN
MEP1A
KRTCAP2
GCNT3
MCM2
MID1IP1
NCSTN
HSPA1B
CDCA5
MFGE8
CNOT11
NSMCE2
C8orf33
PUF60
LYZ
CD24
EPRS
MMP9
DNAJB11
RAP2A
GLA
SNHG6
SLC52A2
SSR2
IRAK1
SAC3D1
SMYD3
MRGBP
GARS
ARHGEF2
ACLY
LOXL4
CTSA
SLC39A1
MANF
LSM4
CCT3
SLC25A39
PLOD3
SRXN1
TEAD2
ATP6AP1
CSTB

ACTA2
CDC25B
PARP1
TMEM106C
MCM6
SMG5
SNRPB
BAIAP2L2
ALG1L
GBA
PSMD4
CNIH4
LMNA
PIK3R2
SAE1
COX7B2
FASN
CKAP4
SIPA1L2
VWF
NEU1
FAM83H
HSPB1
DNMT1
GLMP
ILF2
PTTG1
TUBA1C
COL5A2
HGS
HSP90AB1
MUC13
PTTG3P
RRAGD
SPP1
BRSK1
TOMM40L
CDKN3
S100A10
AURKA
TMEM45B
PEA15
TFRC
GBP2

SQLE
ASPM
NCAPG
LAPTM4B
TKT
CCNB2
COL1A1
LCN2
ACTG2
PAFAH1B3
AKR1C3
SF3B4
DDX39A
LAMC1
FADS1
PRC1
S100P
THY1
UBD
COL4A1
CAP2
CDC20
CCL20
TOP2A
AKR1B10
GPC3
SPINK1

Differentially expressed miRNAs
hsa-miR-224
hsa-miR-10b
hsa-miR-221
solexa-8211-102
hsa-miR-222
hsa-miR-182
hsa-miR-183
hsa-miR-106b
hsa-miR-93
hsa-miR-135a
hsa-miR-18a
hsa-miR-452*:9.1
hsa-miR-96
hsa-miR-500
hsa-miR-452

hsa-miR-324-5p
hsa-miR-551b
hsa-miR-877
hsa-miR-501-5p
hsa-miR-886-5p
hsa-miR-106b*
hsa-miR-454*
hsa-miR-17-5p:9.1
hsa-miR-1180
hsa-miR-1303
hsa-miR-339-3p
hsa-miR-34b*
hsa-miR-7-2*
hsa-miR-934
hsa-miR-499-3p
hsa-miR-1827
HS_151.1
hsa-miR-545
hsa-miR-873
HS_267
HS_220
hsa-miR-558
hsa-miR-548k
HS_130
hsa-miR-569
hsa-miR-488
hsa-miR-921
hsa-miR-133b
hsa-let-7c*
hsa-miR-490-3p
HS_89
hsa-miR-586
HS_43.1
HS_30
hsa-miR-1256
hsa-miR-936
hsa-miR-490-5p
hsa-miR-541*
hsa-miR-1258
HS_144
hsa-miR-600
hsa-miR-545*
HS_62
HS_122.1

hsa-miR-657
hsa-miR-1208
hsa-miR-1324
hsa-miR-1282
hsa-miR-578
hsa-miR-933
HS_84
hsa-miR-548p
hsa-miR-384
HS_133.1
hsa-miR-1179
hsa-miR-100*
hsa-miR-1297
hsa-miR-302d
hsa-miR-559
hsa-miR-576-3p
hsa-miR-1226*
HS_251.1
hsa-miR-187*
HS_65
hsa-miR-602
hsa-miR-542-5p
hsa-miR-554
HS_153
HS_90
hsa-let-7e*
hsa-miR-138
hsa-miR-1294
hsa-miR-30c-2*
HS_262.1
hsa-miR-19b-2*
hsa-miR-573
hsa-miR-219-5p
hsa-miR-101*
HS_199
HS_93
hsa-miR-1184
hsa-miR-125a-3p
HS_252.1
hsa-miR-641
hsa-miR-1272
hsa-miR-23a*
hsa-miR-99a*
hsa-miR-924

hsa-miR-223*
hsa-miR-1299
hsa-miR-367*
hsa-miR-27a*
hsa-miR-133a
HS_203
hsa-miR-195*
hsa-miR-1468
hsa-miR-298
hsa-miR-610
hsa-miR-1
hsa-miR-372
hsa-miR-502-5p
hsa-miR-1207-5p
hsa-miR-609
hsa-miR-592
hsa-miR-890
HS_113
hsa-miR-144:9.1
HS_114
HS_176
hsa-miR-651
hsa-miR-424*
hsa-miR-1284
hsa-miR-181a*
hsa-miR-326
hsa-miR-24-1*
hsa-miR-144
hsa-miR-192*
HS_268
HS_71.1
hsa-miR-1254
HS_38.1
hsa-miR-193b*
HS_81
hsa-miR-26a-2*
HS_150
HS_97
hsa-miR-582-5p
hsa-miR-551a
hsa-miR-125b-2*
HS_244
hsa-miR-17*
hsa-miR-29b-1*

HS_139
HS_152
hsa-miR-139-3p
HS_32
hsa-miR-10a*
hsa-let-7a*
hsa-miR-1247
hsa-miR-940
hsa-miR-374a*
hsa-miR-548b-5p
HS_275
hsa-miR-181a-2*
hsa-miR-335*
hsa-miR-455-5p
hsa-miR-143*
hsa-miR-26b*
HS_209.1
hsa-miR-214*
hsa-miR-203
hsa-miR-624*
HS_239
hsa-miR-378*
hsa-miR-511
hsa-let-7b*
hsa-miR-542-3p
hsa-miR-148a
hsa-miR-615-3p
hsa-miR-194*
hsa-miR-189:9.1
hsa-miR-129-5p
hsa-miR-450b-5p
hsa-miR-340
hsa-miR-29c*
hsa-miR-22*
hsa-miR-144*
solexa-9029-92
hsa-miR-204
hsa-miR-126
hsa-miR-497
hsa-miR-576-5p
hsa-miR-145
hsa-miR-499-5p
hsa-miR-145*
hsa-miR-130a

hsa-miR-450a
hsa-miR-22
hsa-miR-29c
hsa-miR-335
hsa-miR-505
hsa-miR-30a
hsa-miR-30e
hsa-miR-486-5p
hsa-miR-30a*
hsa-miR-101
hsa-let-7c
hsa-miR-451
hsa-miR-125b
hsa-miR-378
hsa-miR-195
hsa-miR-424
hsa-miR-10a
hsa-miR-122*
hsa-miR-100
hsa-miR-214
hsa-miR-139-5p
hsa-miR-99a
hsa-miR-199a-5p
hsa-miR-199a*:9.1
