# Supplementary material for: Identification of key miRNAs in the progression of hepatocellular carcinoma using an integrated bioinformatics approach
Source: PeerJ. 2020 May 6;8:e9000. doi: 10.7717/peerj.9000 (PMC7210814; doi:10.7717/peerj.9000)
Supplement: Supplemental Information 4 [file peerj-08-9000-s004.pdf]

Table S2:
Genes in ME

HAMP
FCN3
CLEC1B
LCAT
IGFALS
CLEC4G
DNASE1L3
SLC25A47
SRD5A2
KBTBD11
RND3
DBH
CDHR2
TCIM
CNDP1
CHST4
TMEM27
HAO2
DCN
INMT
VIPR1
CYP39A1
ST3GAL6
MARCO
OIT3
CETP
ZGPAT
ATOH8
ECM1
AKR1C3
UBD
CAP2
GPC3
