# Supplementary material for: Identification of key miRNAs in the progression of hepatocellular carcinoma using an integrated bioinformatics approach
Source: PeerJ. 2020 May 6;8:e9000. doi: 10.7717/peerj.9000 (PMC7210814; doi:10.7717/peerj.9000)
Supplement: Supplemental Information 5 [file peerj-08-9000-s005.pdf]

**Table S3:**

| mRNAs binding TFs (from JASPAR) |
| --- |
| RUNX1 |
| TFAP2A |
| NR2F1 |
| CREB1 |
| E2F1 |
| NFIL3 |
| ELK1 |
| FOXF2 |
| FOXD1 |
| FOXC1 |
| FOXL1 |
| GATA2 |
| GATA3 |
| FOXI1 |
| HLF |
| NHLH1 |
| IRF1 |
| IRF2 |
| MEF2A |
| MZF1_1-4 |
| MZF1_5-13 |
| MAX |
| MYC::MAX |
| GABPA |
| PPARG::RXRA |
| PPARG |
| Pax6 |
| PBX1 |
| RORA_1 |
| RORA_2 |
| RREB1 |
| RXRA::VDR |
| ELK4 |
| SOX9 |
| SP1 |
| SPI1 |
| SPIB |
| SRF |
| SRY |
| TEAD1 |
| TAL1::TCF3 |
| USF1 |

YY1
ETS1
REL
NFKB1
TP53
RELA
NR1H2::RXRA
TLX1::NFIC
NKX3-1
ZNF354C
HINFP
BRCA1
STAT1
REST
CTCF
FOXA1
EWSR1-FLI1
REST
STAT1
ESR1
NFE2L2
INSM1
RXR::RAR_DR5
NFIC
PLAG1
SPI1
ESR2
FOXA1
BATF::JUN
CDX2
CEBPB
DUX4
E2F4
E2F6
ELF1
FLI1
FOS
FOSL1
FOSL2
FOXH1
FOXP1
HNF4G
HSF1
JUN

JUN (var.2)
JUNB
JUND
JUND (var.2)
MAFF
MAFK
MEF2C
NFE2::MAF
NFYB
NR2C2
NRF1
POU2F2
PRDM1
RFX5
RUNX2
SMAD2::SMAD3::SMAD4
SP2
STAT2::STAT1
TCF7L2
TFAP2C
TP63
USF2
ZBTB33
ZNF263
AR
CEBPA
E2F1
EBF1
EGR1
ELK4
ESR2
FOXA1
GATA2
GATA3
HNF4A
IRF1
MAX
MEF2A
NFKB1
NFYA
PAX5
SP1
SRF
STAT1

STAT3
TAL1::GATA1
TFAP2A
TP53
USF1
YY1
ZEB1
ESRRA
FOXP2
SREBF1
SREBF2
THAP1
EHF
KLF5
RFX2
