# Supplementary material for: Identification of key miRNAs in the progression of hepatocellular carcinoma using an integrated bioinformatics approach
Source: PeerJ. 2020 May 6;8:e9000. doi: 10.7717/peerj.9000 (PMC7210814; doi:10.7717/peerj.9000)
Supplement: Supplemental Information 6 [file peerj-08-9000-s006.pdf]

Table S4:
TOP 5% mRNA-TF pairs

mRNA	TF
VIPR1	ZNF263
DBH	ZNF263
DCN	ZNF263
INMT	PPARG
CAP2	ZNF263
DBH	EWSR1-FLI1
VIPR1	IRF1
ST3GAL6	MEF2C
FCN3	STAT2::STAT1
RND3	ZNF263
CHST4	TLX1::NFIC
DCN	IRF1
ECM1	SP2
FCN3	IRF1
HAO2	MAFF
HAO2	HSF1
ST3GAL6	MEF2A
KBTBD11	PRDM1
ST3GAL6	ZNF263
KBTBD11	IRF1
MARCO	PRDM1
ST3GAL6	EWSR1-FLI1
HAO2	CTCF
LCAT	RREB1
CYP39A1	RFX2
DCN	EWSR1-FLI1
FCN3	STAT1
SLC25A47	MEF2A
DNASE1L3	FOXP1
IGFALS	SP2
HAO2	MAFK
VIPR1	SP2
AKR1C3	MAFF
CNDP1	EGR1
GPC3	PLAG1
MARCO	ESRRA
CDHR2	ESRRA
GPC3	NFYA
UBD	HNF4A
IGFALS	SP1
CETP	HSF1

CETP	REST
CDHR2	FOXP1
KBTBD11	NRF1
GPC3	SP2
DCN	SRF
UBD	NFKB1
CETP	MEF2A
CDHR2	ZNF263
LCAT	RFX2
SLC25A47	NR2C2
VIPR1	ELF1
RND3	FOXP1
UBD	HNF4G
RND3	TP63
OIT3	IRF1
ATOH8	HNF4A
ATOH8	ZNF263
CLEC1B	ZNF263
FCN3	PRDM1
CDHR2	EBF1
CLEC1B	GATA2
UBD	RELA
IGFALS	TEAD1
ST3GAL6	PAX5
CDHR2	STAT3
ATOH8	HNF4G
VIPR1	RFX5
INMT	ESR2
DCN	PRDM1
AKR1C3	MAFK
OIT3	TAL1::GATA1
CYP39A1	STAT2::STAT1
KBTBD11	SP2
AKR1C3	JUND (var.2)
CHST4	TEAD1
KBTBD11	EGR1
RND3	FOXP2
DNASE1L3	FOXP2
INMT	FOXP2
CETP	TAL1::GATA1
INMT	NFKB1
DNASE1L3	FOXA1
ZGPAT	SREBF2
DNASE1L3	IRF1

AKR1C3	TCF7L2
UBD	NR2C2
KBTBD11	TAL1::TCF3
CETP	MEF2C
INMT	FOXA1
CYP39A1	SP2
CDHR2	RREB1
CHST4	EGR1
CDHR2	CDX2
ST3GAL6	CEBPA
INMT	HNF4A
ZGPAT	ZNF263
MARCO	FLI1
SRD5A2	RREB1
CHST4	GATA2
VIPR1	EBF1
CETP	SP2
KBTBD11	FOXP1
SRD5A2	PRDM1
INMT	FOXP1
ECM1	TFAP2C
LCAT	EGR1
CLEC4G	HSF1
UBD	TFAP2A
INMT	FOXH1
ZGPAT	SREBF1
ATOH8	NR2F1
UBD	STAT1
CHST4	IRF1
CDHR2	STAT1
OIT3	FOXP1
ZGPAT	STAT2::STAT1
CLEC4G	NFYB
OIT3	STAT1
ZGPAT	IRF1
ZGPAT	SP2
ZGPAT	NFKB1
VIPR1	IRF2
KBTBD11	FLI1
CYP39A1	EGR1
GPC3	NR2C2
DCN	STAT3
ECM1	SP1
VIPR1	SP1

CLEC4G	FOXP1
GPC3	ZNF263
GPC3	INSM1
UBD	TFAP2C
DCN	FOXP2
HAMP	SP1
CLEC4G	RORA_1
VIPR1	YY1
ST3GAL6	E2F6
TCIM	IRF1
CYP39A1	SP1
ST3GAL6	NFE2L2
SLC25A47	SP1
ZGPAT	SP1
VIPR1	EWSR1-FLI1
HAMP	PAX5
KBTBD11	RFX5
LCAT	SP2
CLEC1B	STAT1
ZGPAT	RELA
ST3GAL6	ESR1
DNASE1L3	DUX4
VIPR1	TLX1::NFIC
SLC25A47	E2F4
MARCO	TP53
SRD5A2	ZNF263
GPC3	IRF1
FCN3	EBF1
IGFALS	KLF5
VIPR1	KLF5
GPC3	KLF5
LCAT	CTCF
CDHR2	NR2C2
CLEC4G	TFAP2C
ATOH8	ESRRA
LCAT	KLF5
HAMP	KLF5
CDHR2	ESR1
VIPR1	EGR1
SLC25A47	USF1
CNDP1	USF1
DNASE1L3	CEBPB
KBTBD11	CEBPB
OIT3	CDX2

MARCO	YY1
IGFALS	EGR1
ATOH8	RREB1
ST3GAL6	STAT3
CAP2	STAT1
DCN	FOSL2
IGFALS	PLAG1
ST3GAL6	E2F1
DCN	FOXP1
SRD5A2	AR
VIPR1	FLI1
ZGPAT	ESRRA
LCAT	BATF::JUN
ZGPAT	MAFF
AKR1C3	USF2
ST3GAL6	FOS
LCAT	PLAG1
LCAT	FOS
INMT	MEF2C
ST3GAL6	E2F4
CDHR2	FOXP2
CLEC4G	FOXP2
CHST4	PRDM1
SLC25A47	E2F1
MARCO	ELF1
CYP39A1	NFYB
CHST4	ZNF263
VIPR1	DUX4
DCN	JUNB
CYP39A1	NFYA
INMT	NFYB
ST3GAL6	FOXI1
CLEC4G	SMAD2::SMAD3::SMAD4
DCN	FOXI1
CNDP1	HSF1
ATOH8	SP1
LCAT	FOSL2
INMT	RELA
DCN	FOSL1
IGFALS	FOXA1
UBD	MAFK
DCN	JUN (var.2)
CNDP1	ZNF263
ATOH8	PRDM1

ATOH8	ZEB1
KBTBD11	ZEB1
DBH	ZEB1
DCN	ZEB1
IGFALS	ZEB1
ATOH8	E2F4
GPC3	E2F4
MARCO	ZNF263
FCN3	FOXP1
UBD	PRDM1
AKR1C3	FOXP1
DNASE1L3	MAX
SLC25A47	E2F6
IGFALS	ESR1
CLEC1B	CDX2
RND3	FOXA1
CLEC4G	NFYA
CDHR2	SP2
ST3GAL6	CEBPB
HAO2	SP1
CLEC4G	STAT1
DCN	BATF::JUN
AKR1C3	IRF1
UBD	USF2
AKR1C3	TAL1::TCF3
FCN3	CTCF
CLEC4G	ZBTB33
CDHR2	RXR::RAR_DR5
OIT3	FOXI1
CDHR2	ESR2
ST3GAL6	MAFF
CLEC1B	TEAD1
CHST4	TFAP2A
DCN	JUND (var.2)
CYP39A1	FOXF2
MARCO	IRF1
DBH	KLF5
SLC25A47	KLF5
ZGPAT	KLF5
