# Supplementary material for: Identification of key miRNAs in the progression of hepatocellular carcinoma using an integrated bioinformatics approach
Source: PeerJ. 2020 May 6;8:e9000. doi: 10.7717/peerj.9000 (PMC7210814; doi:10.7717/peerj.9000)
Supplement: Supplemental Information 7 [file peerj-08-9000-s007.pdf]

Table S5:
Top 100 miRNA-TF pairs

TF	miRNA
AML1	hsa-miR-148a
AML1	hsa-miR-302d
ATF6	hsa-miR-214
C-REL	hsa-miR-1
C-REL	hsa-miR-133a
CHOP-C/EBPALPHA	hsa-miR-130a
CdxA	hsa-miR-101
CdxA	hsa-miR-125b
EGR	hsa-miR-93
EGR	hsa-miR-106b
ELF-1	hsa-miR-133b
ELF-1	hsa-miR-214
ER	hsa-miR-1
ER	hsa-miR-101
ER	hsa-miR-96
ER	hsa-miR-144
ER	hsa-miR-183
ER	hsa-miR-133a
ER	hsa-miR-182
ER	hsa-miR-130a
ETS	hsa-miR-214
FREAC-4	hsa-miR-214
GABP	hsa-miR-130a
HIF-1	hsa-miR-214
HNF-1	hsa-miR-144
HNF-3	hsa-miR-135a
HOXA7	hsa-miR-96
HOXA7	hsa-miR-183
HOXA7	hsa-miR-182
HSF2	hsa-miR-148a
IRF	hsa-miR-101
LEF1	hsa-miR-135a
LEF1	hsa-miR-138
MEF-2	hsa-miR-1
MEIS1	hsa-miR-101
MEIS1	hsa-miR-125b
MEIS1	hsa-miR-214
NCX	hsa-miR-101
NCX	hsa-miR-542-3p
NCX	hsa-miR-125b

NF-AT	hsa-miR-148a
NF-Y	hsa-miR-138
NF-Y	hsa-miR-93
NF-Y	hsa-miR-125b
NF-Y	hsa-miR-106b
OCTAMER	hsa-miR-125b
PAX-4	hsa-miR-148a
PAX-4	hsa-miR-125b
PEA3	hsa-miR-497
PEA3	hsa-miR-195
PEA3	hsa-miR-214
POU3F2	hsa-miR-101
RFX	hsa-miR-148a
RORALPHA2	hsa-miR-133a
SF-1	hsa-miR-133a
SOX-5	hsa-miR-302d
SOX-5	hsa-miR-138
SOX-5	hsa-miR-125b
SP-1	hsa-miR-542-3p
SRF	hsa-miR-93
SRF	hsa-miR-214
SRF	hsa-miR-125b
SRF	hsa-miR-106b
SRY	hsa-miR-222
SRY	hsa-miR-221
STAT1	hsa-miR-130a
TEF	hsa-miR-10a
YY1	hsa-miR-148a
YY1	hsa-miR-101
YY1	hsa-miR-497
YY1	hsa-miR-195
YY1	hsa-miR-125b
C-REL	hsa-miR-203
CHX10	hsa-miR-125b
CdxA	hsa-miR-100
EGR	hsa-miR-203
HOXA4	hsa-miR-125b
IRF-7	hsa-miR-133b
IRF	hsa-miR-203
IRF	hsa-miR-125b
IRF	hsa-miR-100
LEF1	hsa-miR-126
LEF1	hsa-miR-203
LEF1	hsa-miR-10a

MEF-2	hsa-miR-133a
MEIS1	hsa-miR-99a
MEIS1	hsa-miR-100
MYOD	hsa-miR-203
NF-Y	hsa-miR-99a
NKX2-5	hsa-miR-505
PBX-1	hsa-miR-135a
PEA3	hsa-miR-133b
RORALPHA2	hsa-miR-125b
SOX-5	hsa-miR-133b
SOX-5	hsa-miR-100
SRF	hsa-miR-100
T3R	hsa-miR-505
TCF-4	hsa-miR-126
TCF-4	hsa-miR-10a
YY1	hsa-miR-100
